# A52 SEX DIFFERENCES IN POST-INFLAMMATORY VISCERAL PAIN IN A MOUSE MODEL OF INFLAMMATORY BOWEL DISEASE (IBD)

**DOI:** 10.1093/jcag/gwac036.052

**Published:** 2023-03-07

**Authors:** M Arzamendi, A Shute, R Chan, M Defaye, C Altier, K A Sharkey, M B Geuking, Y Nasser

**Affiliations:** 1 Department of Medicine, Cumming School of Medicine; 2 Snyder Institute for Chronic Diseases, Cumming School of Medicine; 3 Department of Physiology and Pharmacology, Cumming School of Medicine,; 4 Alberta Children’s Hospital Research Institute; 5 Hotchkiss Brain Institute, Cumming School of Medicine; 6 Department of Microbiology, Immunology and Infectious Diseases, Cumming School of Medicine, University of Calgary, Calgary, Canada

## Abstract

**Background:**

Inflammatory bowel diseases (IBD) are chronic inflammatory disorders of the gastrointestinal tract. Despite achieving endoscopic remission, up to 50% of IBD patients continue to experience chronic abdominal pain, with female patients displaying an increased prevalence. The reason underlying these differences in pain perception is unknown, but the influence of sex hormones represents an important biological source for variability in pain sensitivity. To date, few studies have examined sex differences in chronic visceral pain in IBD.

**Purpose:**

Examine sex-specific differences in post-inflammatory chronic visceral pain in IBD.

**Method:**

We used the post-inflammatory DSS mouse model of chronic visceral pain. Ovariectomy was performed to study the effects of estrogen deficiency; sham surgery was performed as a control. Male, cycling female and ovariectomized female mice were given 2.5% DSS for five days and allowed to recover for 5 weeks. Somatic pain was evaluated using the hot plate and von Frey hair tests. Visceral pain was evaluated using the visceral motor reflex (VMR) to colorectal distension five weeks after DSS treatment. Visceral and somatic pain testing in cycling females was performed in diestrus.

**Result(s):**

Male, cycling female and ovariectomized female mice given DSS initially lost weight when compared to controls (p<0.0001). Cycling females displayed significantly decreased colitis severity when compared to males [Disease Activity Index at Day 12: 1.41 ± 0.41 cycling females, n=12; 4.41 ± 0.31 males, n=12; p<0.001] but increased severity compared to ovariectomized females [Disease Activity Index at Day 12: 2.0 ± 0.41 ovariectomized females, n=13; 4.77 ± 0.6 cycling females, n=13, p=0.0005]. Increased visceral hypersensitivity was seen in post-inflammatory cycling females compared to post-inflammatory males [VMR at 60mmHg, post-inflammatory cycling females 0.10 ± 0.016, n=10; post-inflammatory males 0.07 ± 0.007, n=10; p=0.032] and post-inflammatory ovariectomized females [VMR at 60mmHg, post-inflammatory sham females 0.072 ± 0.005, n=8; post-inflammatory ovariectomized females 0.047 ± 0.005, n=8; p=0.019]. Thermal hyperalgesia and mechanical allodynia were similar across all groups.

**Conclusion(s):**

These data suggest that estrogen plays an important role in the severity of colitis severity and post-inflammatory visceral pain. Understanding sex-specific differences in post-inflammatory visceral pain in IBD may allow us to define novel therapeutic approaches for IBD patients.

**Disclosure of Interest:**

None Declared

